# Fracture of Rotary Instruments in Third Molar Extraction: Evidence from a Scoping Review

**DOI:** 10.3390/clinpract16020033

**Published:** 2026-02-02

**Authors:** Luca Gentili, Roberto Fontanella, Marco Messi, Cosimo Galletti, Roberto Lo Giudice, Francesco Puleio

**Affiliations:** 1 Department of Odontostomatology, Carlo Urbani Hospital, 60036 Jesi, Italy; luca.gentili@sanita.marche.it (L.G.); marco.messi@sanita.marche.it (M.M.); 2 School of Dentistry, Polytechnic University of Marche, 60121 Ancona, Italy; roberto.fontanella.19@gmail.com; 3 Faculty of Medicine and Surgery, Kore University of Enna, 94100 Enna, Italy; cosimo.galletti@unikore.it; 4 Department of Biomedical and Dental Sciences and Morphofunctional Imaging, Messina University, 98122 Messina, Italy; francesco.puleio@unime.it

**Keywords:** third molar, surgery, rotary instruments, bur fracture, iatrogenic complications, mandible, surgical complications, Odontectomy

## Abstract

Background: Rotary instrument fracture during third molar extraction is rare but clinically relevant, presenting diagnostic and therapeutic challenges. Aim: This scoping review summarizes available evidence on bur breakage and displacement during third molar surgery, focusing on causes, clinical manifestations, and management strategies. Materials and Methods: A systematic search of PubMed, Virtual Health Library, and Google Scholar was conducted for studies published from January 2008 to March 2025 reporting rotary instrument fracture during third molar extraction. Extracted data were qualitatively analyzed. Results: Eight studies reporting eleven clinical cases were included. All fractures occurred during mandibular third molar extractions. Pain was the most frequent symptom (45%), followed by swelling (27%) and trismus (18%). Management varied from immediate surgical retrieval to conservative observation. Conclusions: Although uncommon, rotary bur fracture during third molar extraction requires preventive attention and accurate reporting. Adherence to manufacturer recommendations, single-use bur policies, and adequate irrigation should be considered. Prospective multicenter and mechanical studies are needed to establish standardized management protocols.

## 1. Introduction

Third molar extraction is one of the most frequently performed procedures in oral and maxillofacial surgery, indicated for prophylactic, orthodontic, and pathological reasons. Despite its routine nature, this procedure is associated with a wide spectrum of intraoperative and postoperative complications, and thorough knowledge of potential adverse events is essential to minimize morbidity and improve patient outcomes [1,2].

Among the less commonly reported intraoperative complications is the fracture of rotary burs during osteotomy or tooth sectioning. Although rarely documented, this event may create diagnostic and therapeutic challenges and carries clinically relevant medico-legal implications [2,3]. The etiology of rotary bur fracture appears multifactorial and may involve excessive torsional loading, cyclic fatigue, wear-related loss of cutting efficiency, corrosion phenomena and weakening at the shank–head junction, as well as operator- and procedure-related variables such as inadequate irrigation and inappropriate use or reuse beyond the recommended lifespan [3,4,5,6].

Several preventive measures have been proposed to reduce the risk of rotary instrument failure during third molar surgery, including careful inspection of the bur before and after use, avoidance of overuse, adherence to manufacturer recommendations and/or single-use protocols when applicable, appropriate torque/speed control, and adequate irrigation during bone removal to limit heat generation and facilitate debris clearance [3,4,7]. When fracture occurs, the detached fragment may remain at the surgical site or become displaced into adjacent anatomical spaces, potentially resulting in pain, swelling, trismus, infection, or neurological symptoms depending on its location and proximity to vital structures [3,4].

In some circumstances, fragments may become embedded or encapsulated within bone or soft tissues, complicating delayed retrieval and increasing the risk of persistent symptoms [3,4]. Clinicians must therefore decide between immediate surgical retrieval and a conservative strategy based on symptoms, fragment location, and procedural risk. Transparent communication with the patient and appropriate documentation of the iatrogenic event and management plan are essential components of correct clinical practice and medico-legal protection [3,8].

Although postoperative complications of third molar surgery have been widely investigated, intraoperative mishaps are less consistently reported [2,9]. Commonly described intraoperative complications include excessive bleeding, trauma to adjacent teeth or restorations, inadvertent root damage, and oroantral communication; however, epidemiological data regarding the frequency, risk factors, and standardized management of rotary bur fracture during third molar removal are lacking [9].

### Aim

The aim of this scoping review was to map and qualitatively synthesize the existing case-based literature on rotary bur fracture during third molar extraction. Specifically, the review sought to summarize the mechanical and procedural factors reported as possible etiological contributors, describe the spectrum of clinical presentations, and explore the management strategies adopted in published cases. In addition, this review aimed to identify gaps and limitations in the current literature to inform future research and improve clinical awareness [10,11,12,13].

## 2. Materials and Methods

**Figure 1:**
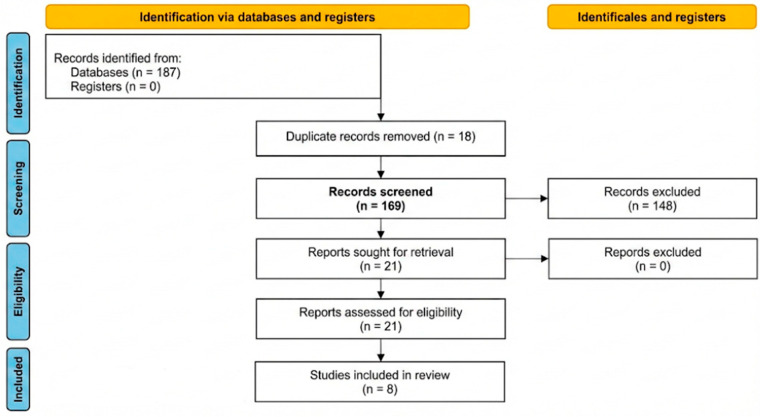
Flow diagram.

This scoping review was conducted in accordance with established methodological frameworks for scoping reviews and reported following the Preferred Reporting Items for Systematic Reviews and Meta-Analyses Extension for Scoping Reviews (PRISMA-ScR) [14,15,16]. A PRISMA 2020 flow diagram was used to illustrate the study selection process, and the PRISMA-ScR checklist was applied to ensure transparent and comprehensive reporting [15] (Figure 1).

The literature search was independently performed by two reviewers using three electronic databases: PubMed/MEDLINE, Virtual Health Library (VHL), and Google Scholar. The search covered studies published from January 2008 to March 2025. The selected time frame was chosen to reflect contemporary surgical techniques, materials, and rotary instrumentation used in third molar surgery [16].

The search strategy combined the following keywords using Boolean operators: dental surgery, third molar, extraction, bur, rotary instruments, breakage, iatrogenic damage, complications, and oral surgery. These terms were applied in multiple two-term combinations to maximize sensitivity and capture rare or heterogeneously reported cases, consistent with scoping review methodology [16,17].

### Inclusion and Exclusion Criteria

Eligibility criteria were defined a priori according to established scoping review recommendations and intentionally designed to be broad [16,17]. Studies were included if they reported fracture of rotary instruments during third molar extraction, provided sufficient clinical information regarding fragment location, management, and outcomes, and were published in English.

Studies were excluded if they did not involve oral or maxillofacial surgery, were unrelated to third molar extraction, consisted exclusively of experimental or laboratory data without clinical correlation, or lacked adequate clinical detail to allow meaningful qualitative analysis [16,17].

Study selection was conducted through title and abstract screening followed by full-text evaluation. Disagreements between reviewers were resolved by discussion and, when necessary, consultation with a third investigator, as recommended for scoping reviews [16].

Given the limited number of eligible studies and the heterogeneity of reported variables, data were analyzed descriptively without quantitative synthesis or meta-analysis, in accordance with accepted scoping review methodology [16,18].

A total of 354 records were initially identified. After duplicate removal and screening, 52 abstracts were reviewed, leading to 8 eligible studies reporting 11 clinical cases. (Table 1) Disagreements between reviewers were resolved through discussion and, if necessary, by consultation with a third investigator. Extracted variables included patient demographics, tooth involved, fragment characteristics, symptoms, treatment timing, anesthesia type, and outcomes. Due to the small sample and heterogeneous reporting, data were analyzed qualitatively. Quantitative results (e.g., symptom frequency, anesthesia type) were summarized descriptively. Data regarding patient characteristics, clinical presentation, fragment location, management approach, and outcomes were extracted as reported in the original case reports. No additional data synthesis or quantitative comparison was performed due to the descriptive nature and heterogeneity of the available evidence, moreover no quantitative synthesis or meta-analysis was feasible. Instead, results were analyzed descriptively to identify patterns and draw clinically relevant conclusions. The selection process is visually summarized in a PRISMA 2020-compliant flow diagram using the tool proposed by Haddaway et al. [15].

## 3. Results

Eight studies met the inclusion criteria, documenting 11 clinical cases of rotary instrument fracture during third molar extraction.

The mean age of patients was 34.3 ± 6.8 years, with a predominance of males (63.6%). All cases occurred in the mandible—six involving the left third molar (tooth 38) and five the right (tooth 48).

Fragments were typically located near the extraction site, with no reports of distant migration. Fragment lengths ranged between 4 and 20 mm. Only one complete bur fracture was documented. Clinical symptoms included pain (45.5%), swelling (27.2%), trismus (18.2%), and limited mouth opening (9%). One case was asymptomatic.

Information concerning the dimensions of the separated fragments was often insufficient. Due to the limited resolution and distortion associated with orthopantomographic imaging, precise measurement was often unfeasible. Only one report described a complete bur breakage, resulting in a retained fragment approximately 20 mm in length. The remaining cases involved partial fractures, though the exact lengths were not consistently specified.

The clinical management strategies following bur breakage were heterogeneous. Two patients underwent immediate surgical removal of the fragment. One patient, who exhibited no symptoms, was managed conservatively with no intervention. In another case, surgery was delayed for five days. The remaining six patients received surgical treatment at unspecified intervals. Among these, three procedures were performed under local anesthesia, while the other three required general anesthesia. The type of anesthesia appeared to correlate with the severity of symptoms or anatomical challenges related to fragment retrieval (Table 2).

These findings represent simple descriptive proportions derived from the included case reports and are reported to illustrate the range of clinical presentations rather than to estimate prevalence or compare outcomes with routine third molar extraction. Due to the small number of cases and the lack of uniformity in reported variables, statistical analysis was not performed. Nonetheless, the data highlight important clinical considerations regarding both the detection and management of this rare but potentially significant intraoperative complication.

## 4. Discussion

The fracture of rotary instruments during third molar surgery represents a rare yet clinically significant intraoperative complication. For consistency, the term “rotary bur” is used throughout the manuscript. When the term “drill” is reported, it reflects the terminology adopted in the original case reports and refers to an equivalent rotary cutting instrument. From an etiological perspective, the available evidence suggests that rotary bur fracture is primarily associated with mechanical and procedural factors, including cyclic fatigue, torsional overload, repeated reuse, corrosion phenomena, and inadequate irrigation. These factors were recurrently described across the included case reports and supporting mechanical studies, although no causal relationships can be inferred due to the nature of the evidence. The limited number of documented cases (*n* = 11) suggests both rarity and possible under-reporting, as asymptomatic or conservatively managed cases may go unrecorded. Most cases occurred during mandibular extractions, consistent with higher bone density and limited surgical access that increase torque stress on the instrument. Furthermore, the predominance of complications on the left side (tooth 38) may be related to operator-handedness and ergonomic constraints, although this remains speculative in the absence of controlled data [2,14].

Rotary burs are typically composed of stainless steel or nickel–chromium alloys, which are subject to cyclic fatigue and torsional overload. Repeated sterilization, corrosion, and microcrack propagation reduce their fatigue resistance, particularly in thinner-shaft designs. Bur geometry, cutting angle, and shaft diameter directly influence torque thresholds and fracturing risk. Factors such as inadequate irrigation, excessive torque, reuse, and microcorrosion further compromise structural integrity. Finite-element and laboratory studies have confirmed that repeated sterilization cycles and extended use markedly diminish fracture resistance in rotary instruments [16,17,18]. Recognizing these mechanical causes is fundamental for both prevention and proper clinical management. From a preventive perspective, rotary bur fracture appears to be influenced by multiple mechanical and procedural factors, including cyclic fatigue, torsional overload, repeated reuse, corrosion phenomena related to sterilization cycles, and inadequate irrigation during osteotomy. Careful inspection of rotary instruments before use, adherence to manufacturer recommendations regarding lifespan, and appropriate control of speed and torque may reduce the risk of instrument failure during third molar surgery. Clinical management of a fractured rotary instrument remains highly variable and should be guided by fragment location, proximity to vital anatomical structures, and patient symptoms. Reported strategies include immediate surgical retrieval, delayed removal under local or general anesthesia, and conservative observation in selected asymptomatic cases. A careful risk–benefit assessment is therefore essential to minimize additional morbidity associated with secondary surgical intervention.

Consistent with the available literature, the present findings confirm that most fractures occur during mandibular third molar extraction, where bone density and limited visibility demand greater torque [2,14]. Previous reviews have described rotary instrument breakage as a multifactorial event involving fatigue failure, defective manufacturing, improper use, corrosion, and repeated utilization without replacement [3]. Our data further suggests that insufficient irrigation and inadequate torque control during osteotomy or odontotomy may play a pivotal role. While Ferreira et al. [3] emphasized the relevance of single-use protocols and visual inspection, very few of the reviewed cases provided technical details sufficient to correlate procedural errors with mechanical failure, highlighting the need for standardized reporting.

The clinical presentation after bur fracture was variable, with pain as the most frequent symptom (45.5%), followed by swelling and trismus. These symptoms, although non-specific, may complicate postoperative diagnosis and delay appropriate management. Interestingly, one case was asymptomatic, reinforcing the need for intraoperative recognition and immediate radiographic evaluation when fracture is suspected. The reported complications were heterogeneous and included pain, swelling, trismus, and asymptomatic presentations. In the absence of standardized outcome measures, these manifestations were descriptively grouped to provide a pragmatic clinical classification based on symptomatology, rather than a severity-based or prognostic categorization [26].

Management strategies reported in the literature are heterogeneous. Some authors opted for immediate surgical retrieval, while others delayed reoperation or chose conservative management. The need for general anesthesia in three of the cases raises concerns about the invasiveness of secondary interventions and underlines the importance of meticulous preoperative planning to minimize such events. The decision to reoperate should balance the risks of leaving a retained fragment against the morbidity of a second surgical procedure—an evaluation requiring sound clinical judgment and patient-specific consideration [8,10]. Decision-making regarding fragment removal was highly variable and context-dependent. Immediate retrieval, delayed surgical removal, and conservative observation were all reported strategies, primarily influenced by fragment location, proximity to vital anatomical structures, and the presence or absence of symptoms [27]. The scoping nature of this review does not allow evaluation of the effectiveness of these strategies but highlights how such decisions are currently approached in clinical practice.

Although the present review focuses on rotary bur fracture, relevant principles can be derived from other surgical domains. For example, A.A., in describing the conservative salvage of an implant associated with a mandibular odontogenic cyst, emphasized the preservation of surrounding anatomical structures during foreign-body retrieval—an approach applicable to fractured burs near vital tissues. Similarly, Puleio, and Rauso et al. [16,17] proposed structured procedural classifications in orthognathic surgery that could inspire standardized frameworks for intraoperative complication management, using and implementing modern procedures and instrumental devices as augmented reality to enhance precision in clinical procedures.

Due to the limited number of available reports and the heterogeneity of the data, no inferential statistical analysis was possible. The descriptive nature of the included literature—mostly case reports or small series—precluded meaningful subgroup comparisons. Moreover, incomplete reporting of critical variables such as fragment size, torque applied, and timing of retrieval prevents deeper analysis. Inclusion of grey literature and non-indexed sources like Google Scholar enhanced comprehensiveness but also introduced variability in peer-review quality. In addition, publication bias and potential under-reporting of intraoperative instrument fracture, partly due to medico-legal concerns, cannot be excluded. Bur fracture is an iatrogenic complication that clinicians may be reluctant to report, leading to an underestimation of its true prevalence in the literature.

Nonetheless, this review provides a structured synthesis of an underreported yet clinically relevant complication, highlighting implications for patient safety, surgical planning, and medico-legal accountability. The inclusion of cases with detailed clinical data strengthens its practical value. The preventive considerations discussed in this review should be interpreted as evidence-informed reflections derived from recurring themes in the published literature and mechanical studies, rather than as evidence-based recommendations supported by comparative or statistical data.

Despite these limitations, the present review underscores the importance of preventive measures such as adherence to single-use protocols, careful torque application, and routine inspection of rotary instruments before use. Increased awareness, meticulous documentation, and standardized multicenter data collection are crucial for developing evidence-based guidelines and reducing the risk of iatrogenic harm in oral and maxillofacial surgery.

## 5. Conclusions

Rotary instrument fracture during third molar extraction is a rare but clinically relevant intraoperative complication. The available evidence, although limited to case reports and small case series, indicates that this event occurs predominantly during mandibular third molar surgery and may be associated with variable clinical presentations, ranging from asymptomatic findings to pain, swelling, and trismus.

Management strategies reported in the literature are heterogeneous and include immediate surgical retrieval, delayed intervention, and conservative observation, depending on fragment location and clinical symptoms. The scarcity and heterogeneity of published data highlight the lack of standardized protocols for the prevention and management of this complication.

This scoping review highlights that rotary bur fracture during third molar extraction is a rare but clinically relevant complication for which the available evidence is limited to isolated case reports and small case series. While the current literature allows a descriptive overview of etiological factors, clinical presentations, and management approaches, it does not support quantitative analysis or assessment of treatment effectiveness. These findings underscore the need for standardized reporting and future prospective studies to generate more robust evidence and guide clinical decision-making.

Future research should focus on standardized mechanical testing of bur fatigue resistance and the development of registry-based reporting systems to enhance traceability and clinical safety.

Improved awareness, prevention, and systematic reporting can significantly reduce the incidence of this rare but potentially serious complication.

## Figures and Tables

**Table 1:** Summary of published cases of rotary instrument fracture during third molar extraction. “Case ID” refers to numbering within the original publication. The terms “drill” and “bur” are used as reported by the authors and denote equivalent rotary instruments.

First Author	Case Number	Patient Age	Sex	Tooth	Fragment Location	Fractured Instrument Type & Size	Signs & Symptoms	Treatment Timing
Kuncai Li [18]	case 1	24	M	48	mandibular bone, distal to 47	4 mm drill tip	/	operated immediately
Kuncai Li [18]	case 2	30	F	38	lingual border of 48 crown on oral mucosa	drill tip	/	operated immediately
Kuncai Li [18]	case 3	25	M	38	fragment incorporated into mandibular bone, initially at 37 then lingually	drill tip	/	operated after 5 days
Fareed Mukram Ali [19]	case 1	35	M	48	mandibular bone, 6.57 mm from superior border of 48 and 7.48 mm from root	bur fragment	not evident	not operated
Ajit Joshi [20]	case 1	43	M	48	mandibular region below third molar	round bur head	pain	operated later
Vimal Kalia [21]	case 1	35	M	48	in submandibular space near mylohyoid crest	drill tip	pain, swelling, limited opening	operated later
Abdulaziz Mahdi [22]	case 1	27	M	38	Mandibular bone adjacent to the extraction site	drill tip	pain	operated later
Shinpei Matsuda [23]	case 1	28	F	36	mandibular body	10 mm bur piece	/	operated later under general anesthesia
Shinpei Matsuda [23]	case 2	35	F	38	under mucosa of oral floor	7 mm bur piece	/	operated later under general anesthesia
Joshi Ramalingam Rajaram [24]	case 1	42	M	48	mandibular bone, distal to 47	9 mm drill tip	pain, swelling, trismus	operated later under general anesthesia
Serhat Jucin [25]	case 1	35	M	48	submandibular space	20 mm flame-shaped bur	pain, swelling, trismus	operated later under local anesthesia

**Table 2:** Symptom distribution and management approaches: Pain (45.5%), Swelling (27.2%), Trismus (18.2%), Asymptomatic (9%). Immediate surgical retrieval was performed in 2 cases, delayed retrieval in 8, and conservative management in 1. Three patients required general anesthesia, while others were treated under local anesthesia.

Symptom	Frequency (%)	Treatment Modality	% of Cases
Pain	45.5	Immediate/Delayed Surgery	81.8
Swelling	27.2	General Anesthesia	27.2
Trismus	18.2	Conservative	9.0
None	9.1	–	–

## Data Availability

The original contributions presented in this study are included in the article. Further inquiries can be directed to the corresponding author.

## References

[1] Sifuentes-Cervantes J.S., Díaz-Ruiz M.A., Fernández-González R., Morales-Velázquez G., Téllez-Girón-Valdés M. (2021). Third molar surgery: Past, present, and the future. Oral Surg. Oral Med. Oral Pathol. Oral Radiol..

[2] Bouloux G.F., Steed M.B., Perciaccante V.J. (2007). Complications of third molar surgery. Oral Maxillofac. Surg. Clin. N. Am..

[3] Ferreira L., Silva J., Oliveira A., Almeida R. (2024). Zekrya bur fracture during extraction: How can this type of complication be avoided? A literature review. J. Oral Health Dent. Sci..

[4] Sánchez-Torres A., Gay-Escoda C., Berini-Aytés L., Camps-Font O. (2020). Patient, radiological, and operative factors associated with surgical difficulty in the extraction of third molars: A systematic review. Int. J. Oral Maxillofac. Surg..

[5] Chuang S.K., Perrott D.H., Susarla S.M., Dodson T.B. (2007). Age as a risk factor for third molar surgery complications. J. Oral Maxillofac. Surg..

[6] Martínez-Rodríguez N., González-Moles M.Á., Morales-García P., Ramos-García P. (2015). Exodoncia en pacientes geriátricos con bifosfonatos. Av. Odontoestomatol..

[7] Halabí D., Escobar J., Muñoz C., Uribe S. (2012). Logistic regression analysis of risk factors for the development of alveolar osteitis. J. Oral Maxillofac. Surg..

[8] Niekrash C., Goupil M.T., Ferneini E.M., Goupil M.T. (2019). Surgical complications. Evidence-Based Oral Surgery: A Clinical Guide for the General Dental Practitioner.

[9] Jaroń A., Trybek G., Grzywacz E., Dobrzyński M., Kaczmarzyk T. (2021). The impact of using Kinesio tape on non-infectious complications after impacted mandibular third molar surgery. Int. J. Environ. Res. Public Health.

[10] Pierse J.E., Dym H., Clarkson E. (2012). Diagnosis and management of common postextraction complications. Dent. Clin. N. Am..

[11] Antunes A.A., Avelar R.L., Martins Neto E.C., Silva W.A., Andrade E.S. (2013). Extensive cervical necrotizing fasciitis of odontogenic origin. J. Craniofac. Surg..

[12] Dym H., Weiss A. (2012). Exodontia: Tips and techniques for better outcomes. Dent. Clin. N. Am..

[13] Barboza S.G., Pereira Y.C.S. (2016). Clasificaciones Winter y Pell-Gregory predictoras del trismo postexodoncia de terceros molares inferiores incluidos. Rev. Odont Mex..

[14] Sisk A.L., Hammer W.B., Shelton D.W., Joy E.D. (1986). Complications following removal of impacted third molars: The role of the experience of the surgeon. J. Oral Maxillofac. Surg..

[15] Haddaway N.R., McGuinness L.A., Pritchard C.C. (2022). PRISMA2020: An R package and Shiny app for producing PRISMA 2020-compliant flow diagrams with interactivity for optimised digital transparency and open synthesis. Campbell Syst. Rev..

[16] Puleio F., Tosco V., Pirri R., Simeone M., Monterubbianesi R., Giudice G.L., Giudice R.L. (2024). Augmented Reality in Dentistry: Enhancing Precision in Clinical Procedures—A Systematic Review. Clin. Pract..

[17] Rauso R., Tartaro G., Nicoletti G.F., Fragola R., Giudice G.L., Santagata M. (2021). Alar cinch sutures in orthognathic surgery: Scoping review and proposal of a classification. Int. J. Oral Maxillofac. Surg..

[18] Li K., Xie B., Chen J., He Y. (2022). Breakage and displacement of the high-speed handpiece bur during impacted mandibular third molar extraction: Three cases. BMC Oral Health.

[19] Ali F.M., Khan M.A., Shtaifi A.E., Namis S.M. (2016). Accidental high-speed handpiece bur buried during surgery of mandibular third molar: A rare case report. MOJ Clin. Med. Case Rep..

[20] Joshi A., Goel M., Gawande M.J. (2017). An unusual accident during a molar extraction: A rare case report—Breakage of surgical instrument, an uncommon intra-operative complication. J. Head Neck Spine Surg..

[21] Kalia V., Kalra G., Singh G., Sharma V. (2015). Localization of broken surgical bur in the submandibular space: Its prevention, retrieval and the role of cone beam computed tomography (CBCT). J. Clin. Case Rep..

[22] Mahdi A.A., Alkharji A.I., Alburayh S.A., Fatani B.A., Alharbi O.A. (2025). Separated surgical instrument during the extraction of a third molar: A case report. Cureus.

[23] Matsuda S., Yoshimura H., Yoshida H., Sano K. (2020). Breakage and migration of a high-speed dental handpiece bur during mandibular third molar extraction: Two case reports. Medicine.

[24] Rajaran J.R., Nazimi A.J., Rajandram R.K. (2017). Iatrogenic displacement of a high-speed bur during third molar removal. BMJ Case Rep..

[25] Yalcin S., Aktas I., Emes Y., Atalay B. (2008). Accidental displacement of a high-speed handpiece bur during mandibular third molar surgery: A case report. Oral Surg. Oral Med. Oral Pathol. Oral Radiol. Endod..

[26] D’Ambrosio F., Di Spirito F., De Caro F., Lanza A., Passarella D., Sbordone L. (2022). Adherence to Antibiotic Prescription of Dental Patients: The Other Side of the Antimicrobial Resistance. Healthcare.

[27] Consorti G., Monarchi G., Mariagrazia Paglianiti Betti E., Balercia P. (2024). Reduction of Post-Surgical Facial Edema Following Bromelain and Coumarin Intake in Traumatology: A Prospective Study with 100 Patients. J. Clin. Med..

